# Serial analysis of coronary artery disease progression by artificial intelligence assisted coronary computed tomography angiography: early clinical experience

**DOI:** 10.1186/s12872-022-02951-9

**Published:** 2022-11-26

**Authors:** Geoffrey W. Cho, Lauren Anderson, Carlos G. Quesada, Robert S. Jennings, James K. Min, James P. Earls, Ronald P. Karlsberg

**Affiliations:** 1 Clinical Faculty in Medicine/Cardiology, UCLA David Geffen School of Medicine, 100 UCLA Medical Plaza, Suite 545, Los Angeles, CA 90095 USA; 2 Cleerly Inc, New York, NY USA; 3 George Washington University, Washington, DC USA; 4 Cedars-Sinai Smidt Heart Institute, Los Angeles, CA USA; 5 Cardiovascular Research Foundation of Southern California, Beverly Hills, CA USA

**Keywords:** Coronary artery disease progression, Coronary computed tomography angiography, Artificial intelligence, Atheroma plaque characteristics, Case report

## Abstract

**Background:**

Studies have shown that quantitative evaluation of coronary artery plaque on Coronary Computed Tomography Angiography (CCTA) can identify patients at risk of cardiac events. Recent demonstration of artificial intelligence (AI) assisted CCTA shows that it allows for evaluation of CAD and plaque characteristics. Based on publications to date, we are the first group to perform AI augmented CCTA serial analysis of changes in coronary plaque characteristics over 13 years. We evaluated whether AI assisted CCTA can accurately assess changes in coronary plaque progression, which has potential clinical prognostic value in CAD management.

**Case presentation:**

51-year-old male with hypertension, hyperlipidemia and family history of myocardial infarction, underwent CCTA exams for anginal symptom evaluation and CAD assessment. 5 CCTAs were performed between 2008 and 2021. Quantitative atherosclerosis plaque characterization (APC) using an AI platform (Cleerly), was performed to assess CAD burden. Total plaque volume (TPV) change-over-time demonstrated decreasing low-density non-calcified plaque (LD-NCP) with increasing overall NCP and calcified-plaque (CP). Examination of individual segments revealed a proximal-LAD lesion with decreasing NCP over-time and increasing CP. In contrast, although the D2/D1/ramus lesions showed increasing stenosis, CP, and total plaque, there were no significant differences in NCP over-time, with stable NCP and increased CP. Remarkably, we also consistently visualized small plaques, which typically readers may interpret as false positives due to artifacts. But in this case, they reappeared each study in the same locations, generally progressing in size and demonstrating expected plaque transformation over-time.

**Conclusions:**

We performed the first AI augmented CCTA based serial analysis of changes in coronary plaque characteristics over 13 years. We were able to consistently assess progression of plaque volumes, stenosis, and APCs with this novel methodology. We found a significant increase in TPV composed of decreasing LD-NCP, and increasing NCP and CP, with variations in the evolution of APCs between vessels. Although the significance of evolving APCs needs to be investigated, this case demonstrates AI-based CCTA analysis can serve as valuable clinical tool to accurately define unique CAD characteristics over time. Prospective trails are needed to assess whether quantification of APCs provides prognostic capabilities to improve clinical care.

**Supplementary Information:**

The online version contains supplementary material available at 10.1186/s12872-022-02951-9.

## Background

Coronary artery disease (CAD) is a widespread global health problem that carries significant cardiovascular morbidity and mortality. Over time, patients frequently develop advanced coronary atherosclerosis due to clustering of traditional atherosclerotic risk factors, such as diabetes, hyperlipidemia, hypertension, etc. Patients with CAD are 8 times more likely to die compared with the general U.S. population, and cardiovascular causes account for > 40% of all deaths [1]. Comorbidity, poor exercise capacity, and a high prevalence of cardiovascular abnormalities limit the diagnostic accuracy of traditional ischemia-driven tests [2]. Coronary computed tomographic angiography (CCTA) represents an established method for the noninvasive detection of CAD and recent developments have allowed for a more accurate quantitative assessment of both the type and volume of coronary atherosclerosis [2]. Recent multicenter studies have shown that quantitative evaluation of coronary artery plaque on CCTA can identify patients at risk of subsequent cardiac events and culprit lesions in patients who subsequently experience a myocardial infarction [2–4]. In addition, recent demonstration of artificial intelligence augmented CCTA shows that it allows for rapid accurate evaluation of CAD and plaque characteristics [5]. Based on all available publications to date, we are the first group to perform artificial intelligence augmented CCTA multi-serial analysis of 5 CT studies to evaluate changes in coronary plaque characteristics over a longer time period of 13 years. Therefore, we aim to assess whether AI assisted CCTA can assess changes in high risk coronary plaque type progression, which has the potential to be of clinical prognostic value leading to improvements in CAD management.

## Case presentation

51-year-old male with hypertension, hyperlipidemia and family history of hypercholesterolemia and early myocardial infarction, underwent serial CCTA exams for anginal symptom evaluation and CAD assessment, which according to the updated 2021 AHA/ACC guidelines regarding use of CCTA, is a Class IA indication to pursue CCTA for exclusion of atherosclerotic plaque and obstructive coronary artery disease (CAD) in intermediate-risk patients with acute chest pain and also for diagnosis of CAD, risk stratification and guiding treatment decisions for patients with stable chest pain who have an intermediate-high risk of obstructive CAD. 5 CCTAs were performed between 2008 and 2021 using various clinically established acquisitions protocols (Siemens 64/Definition, GE Lightspeed VCT/Discovery CT 750, GE Revolution Maxima). Quantitative atherosclerosis plaque characterization (APC) using a semi-automated artificial-intelligence (AI) platform (Cleerly) and FFR-CT (Heartflow), via protocols as defined in the CLARIFY trial [4]. Specifically, CCTA studies were uploaded to and analyzed by FDA-cleared software Cleerly-Labs and automated analysis of CCTA was performed via serial validated convolutional neural network models (VGG 19 network, 3D U-Net, VGG Network Variant) for image quality assessment, coronary segmentation/labeling, lumen evaluation and contour determination, as well as plaque characterization. Plaque volumes were calculated for each lesion, summated to total plaque volume (TPV), and categorized using standard CT criteria for Hounsfield units (HU): non-calcified-plaque (NCP) − 30 to + 350 HU; low-density-HU (LD-NCP) < 30 HU; and calcified-plaques (CP) > 350 HU. Stenosis and remodeling index were calculated by lesion diameter divided by reference diameter.

## Discussion and conclusions

**Fig. 1:**
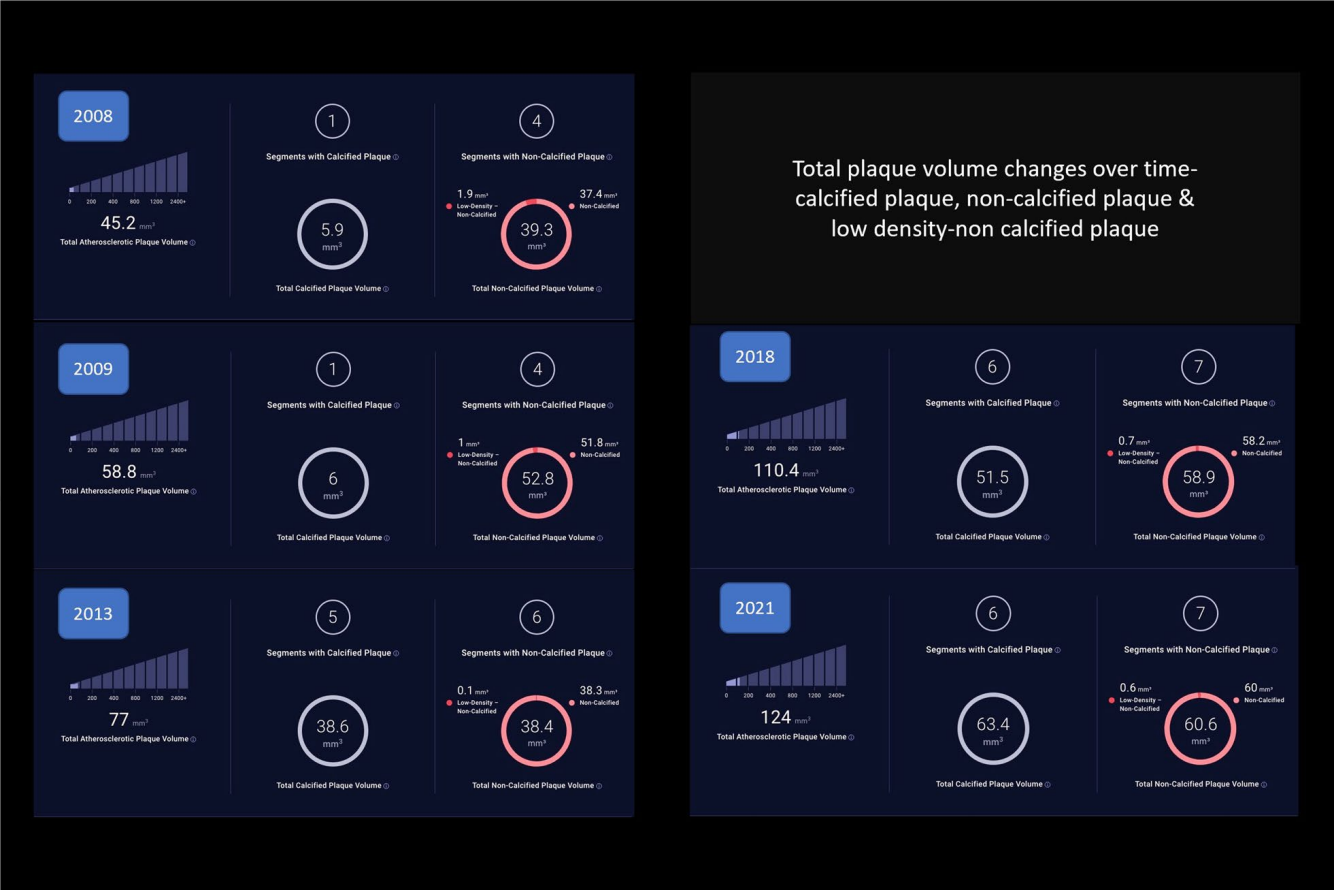
Evolution of atherosclerotic plaque characteristics

**Fig. 2:**
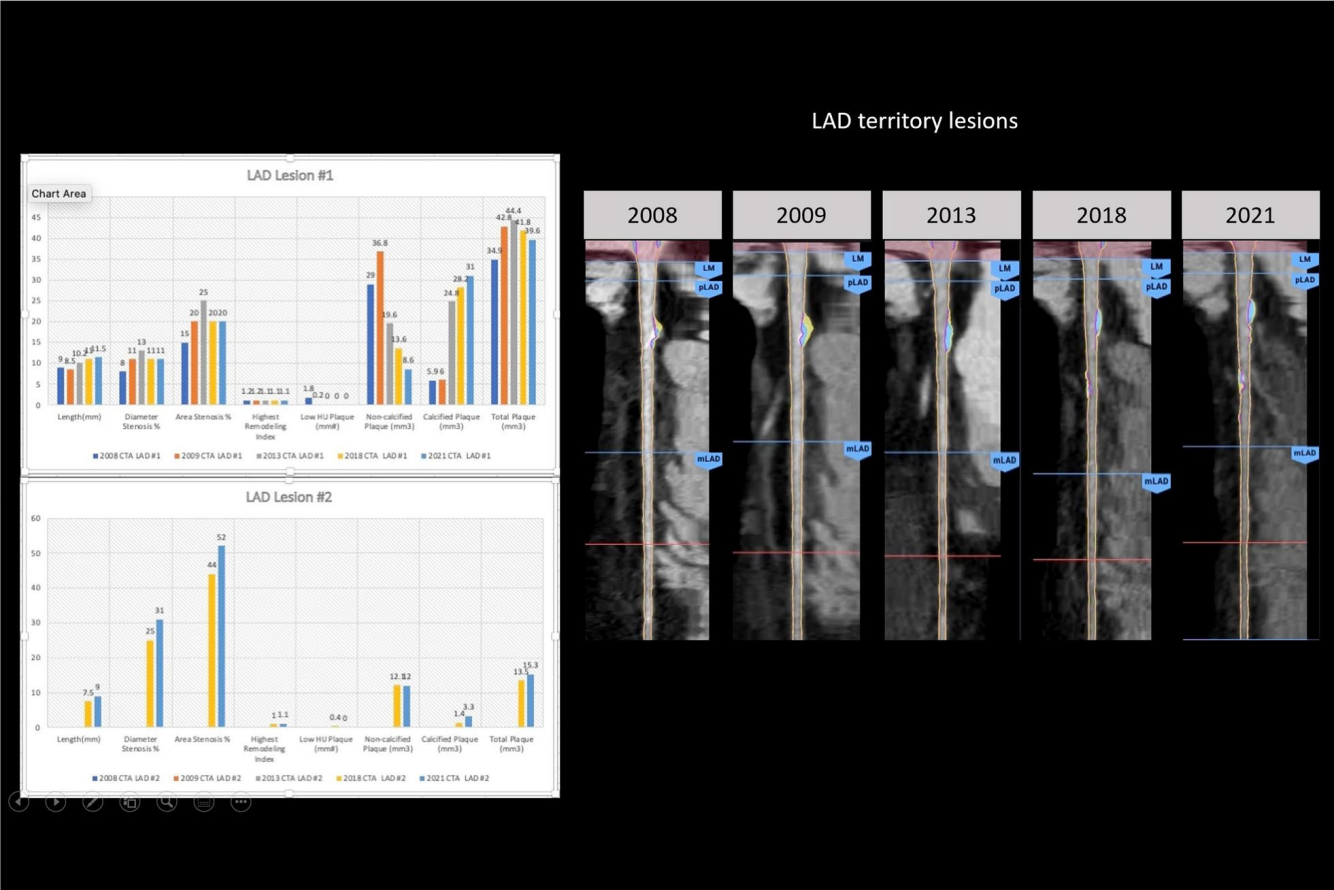
LAD Territory atherosclerotic plaque characteristics

**Fig. 3:**
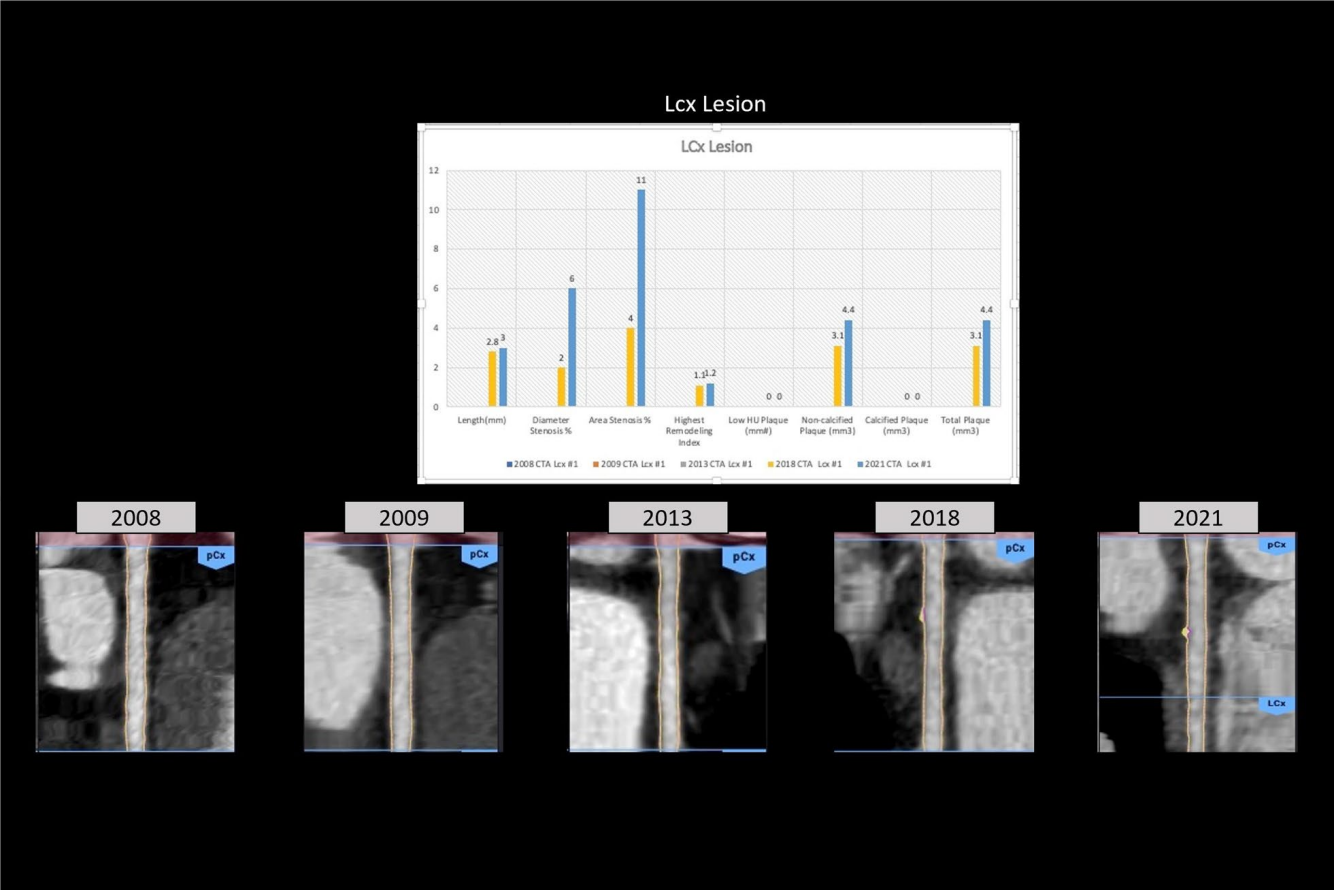
LCX territory atherosclerotic plaque characteristics

11 disease segments were identified for analysis: LAD/diagonals, 5; Circumflex, 2; RCA/PBL, 3; ramus, 1. TPV change-over-time demonstrated decreasing LD-NCP (4-segments[1.9 mm^3^] to 7-segments [0.6 mm^3^]) with increasing overall NCP (4-segments[37.4 mm^3^] to 7-segments [60 mm^3^]) and increasing CP (1-segment [5.9 mm^3^] to 6-segments[63.4 mm^3^]) (Fig. 1). Examination of individual segments revealed a proximal-LAD lesion (increasing length from 9 to 11.5 mm^3^ and stenosis from 15 to 20%) with decreasing NCP over-time (29–8.6 mm^3^) and increasing CP (5.9–31 mm^3^) (Fig. 2). In contrast, although the D2/D1/ramus lesions showed increasing stenosis, CP, and total plaque, there were no significant differences in NCP over-time (D2 length increased from 5.5 to 7.8 mm^3^ with increasing stenosis 8–38%, with stable NCP (6.6 mm^3^) and increased CP (0–12.7 mm^3^) (Additional file 1: Fig S1). Interestingly, the circumflex and more proximal mid-RCA lesions that developed had primarily NCP (Fig. 3 and Additional file 1: Fig. S2), whereas the initial mid-RCA lesion showed an increase of both CP and NCP (Additional file 1: Fig. S2) FFR-CT analysis showed no significant changes except for the D2 lesion which decreased from 0.94 to 0.77 (Additional file 1: Figs. S3 and S4). LDL was optimally managed < 70 mg/dl with a statin and subsequent addition of a PCSK9 inhibitor. Specifically, in terms of timeline, the patient was placed on atorvastatin 80 mg daily starting in 2009, and then evolocumab 140 mg every 2 weeks starting in 2018. 5 serial CCTAs were performed over the course of this study, in 2008, 2009, 2013, 2018 and 2021 (Figs. 1, 2 and 3).

The novelty of this case study is in our following of serial changes in CAD using CCTA (> 2 CCTAs) based AI analysis of coronary plaque characteristics over a period greater than a decade (13 years). The advantage of the AI augmented CCTA software is multifold, including the expedited time to process studies compared to human readers, the potential time/financial benefits saved by not needing human/manual processing of images (ongoing field of study), and the reliability/accuracy of analysis able to be performed at a level equivalent to expert CT level-3 readers [5]. We were able to consistently assess progression of plaque length, volumes, remodeling, stenosis, and APCs with this novel artificial intelligence augmented CCTA methodology. Overall, despite optimal LDL control < 70 mg/dL with use of a statin and PCK9 inhibitor, we found a significant increase in TPV composed of decreasing LD-NCP and increasing NCP and CP. Furthermore, there were variations in the evolution of APCs between vessels, manifesting as changing amounts of LD-NCP, NCP, and CP. Limitations of our study include major restrictions of all case reports with patient numbers being an N of 1, which may lack generalizability and reproducibility. However, recent clinical studies such as the CLARIFY trial, have demonstrated that AI assisted CCTA can accurately and consistently quantify CAD morphology and stenosis. Although the significance of evolving APCs over-time to predict CAD/MACE outcomes needs to be investigated, this case demonstrates AI-based CCTA analysis can serve as valuable clinical tool by accurately defining unique patient CAD characteristics—and future prospective trails are needed to assess whether this ability to further quantify APCs may provide further prognostic capabilities for AI-based CCTA to improve clinical care [4, 5].

## Supplementary Information

**Additional file 1: Figure S1** D1/D2/RI Territory Atherosclerotic Plaque Characteristics. **Figure S2** RCA Territory Atherosclerotic Plaque Characteristics. **Figure S3** FFR-CT Analysis Based on First CCTA. **Figure S4** FFR-CT Analysis Based on Final CCTA.

## Data Availability

The data analyzed for this case report is available upon reasonable request. Authors GWC or RPK can be contacted for data access at gcho@mednet.ucla.edu or Karlsberg@cvmg.com, respectively.

## Abbreviations

AI: Artificial intelligence
APCs: Atheroma plaque characteristics
CAD: Coronary artery disease
CCTA: Coronary computed tomography angiography
CP: Calcified-plaques
FFR: Fractional flow reserve
HU: Hounsfield units
LD-NCP: Low-density non-calcified-plaque
MACE: Major adverse cardiovascular event
NCP: Non-calcified-plaque
TPV: Total plaque volume
