# Supplementary material for: Serial analysis of coronary artery disease progression by artificial intelligence assisted coronary computed tomography angiography: early clinical experience
Source: BMC Cardiovasc Disord. 2022 Nov 26;22:506. doi: 10.1186/s12872-022-02951-9 (PMC9701371; doi:10.1186/s12872-022-02951-9)

# Supplemental Figure 1 – D1/D2/RI Territory Atherosclerotic Plaque Characteristics

#
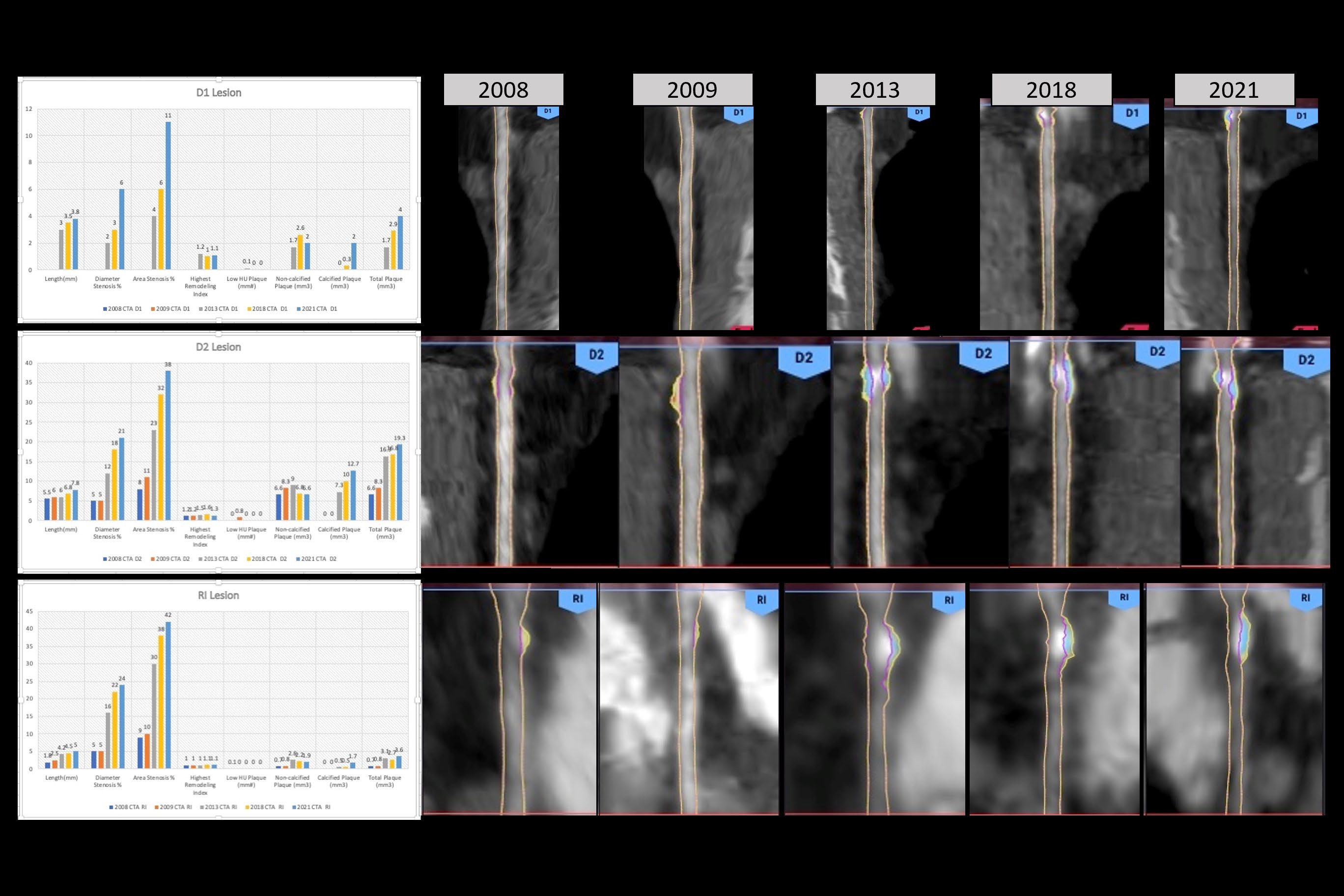


# Supplemental Figure 2 - RCA Territory Atherosclerotic Plaque Characteristics


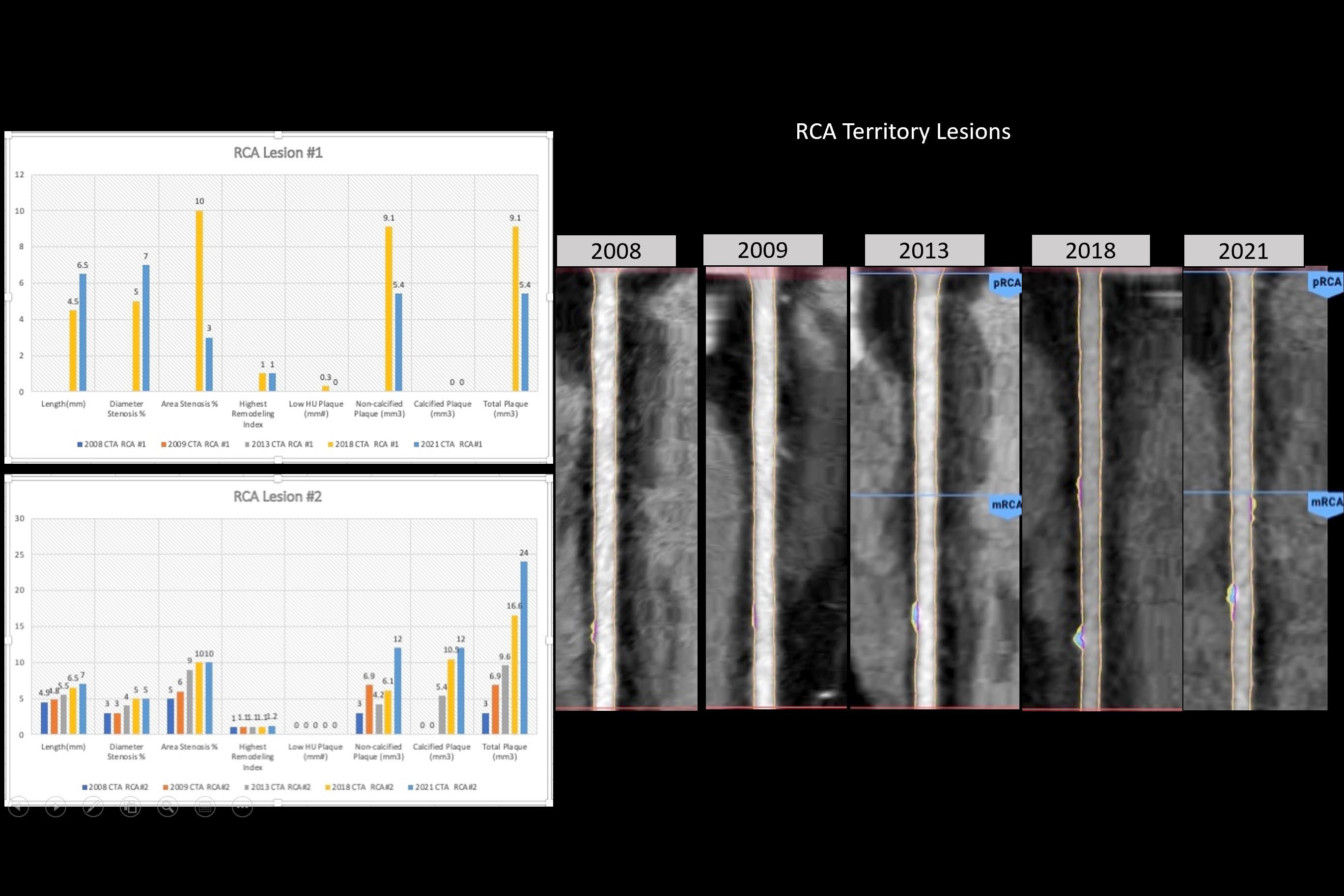


**Supplemental Figure 3 – FFR-CT Analysis Based on First CCTA**
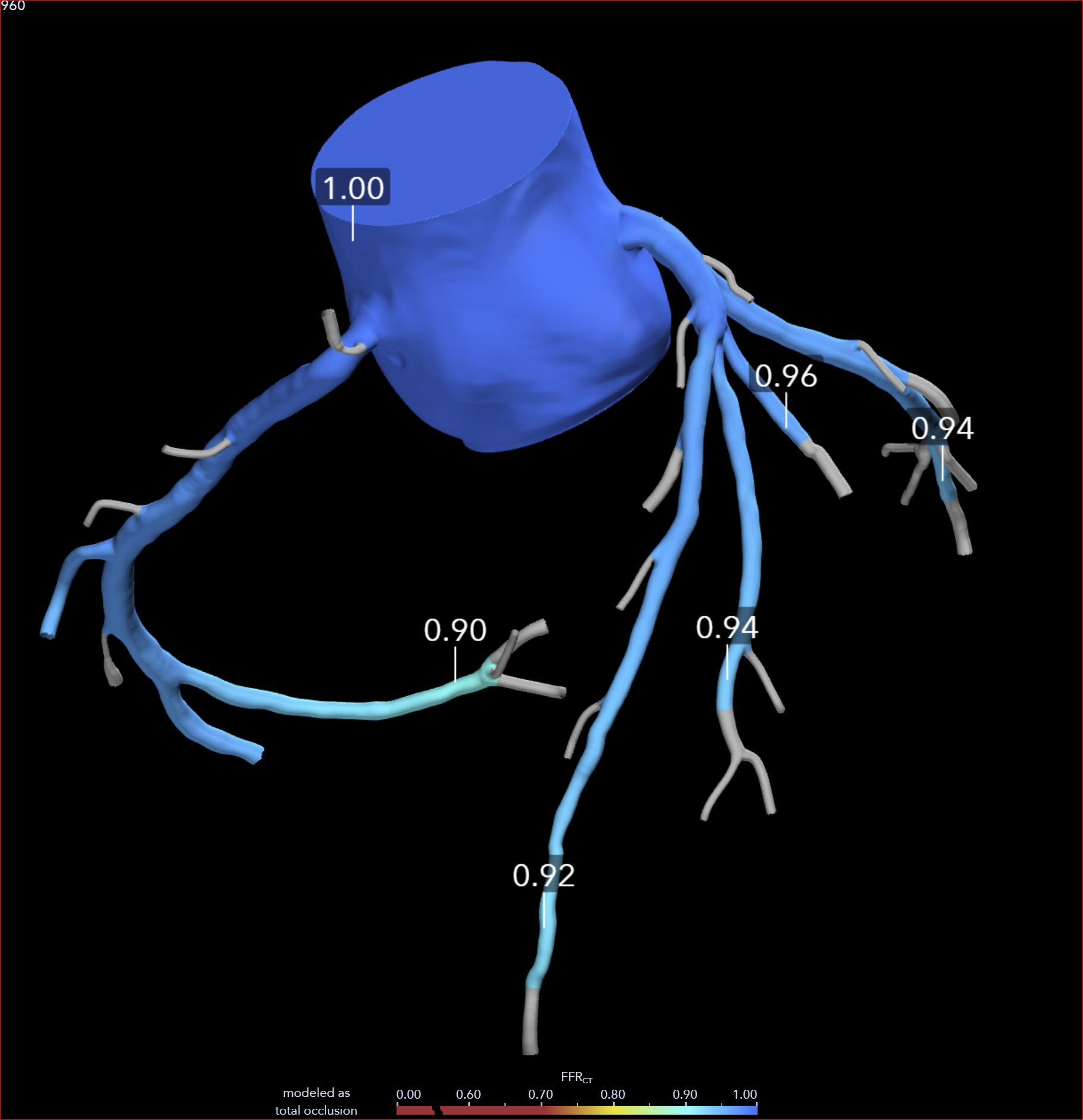


**Supplemental Figure 4 - FFR-CT Analysis Based on Final CCTA**
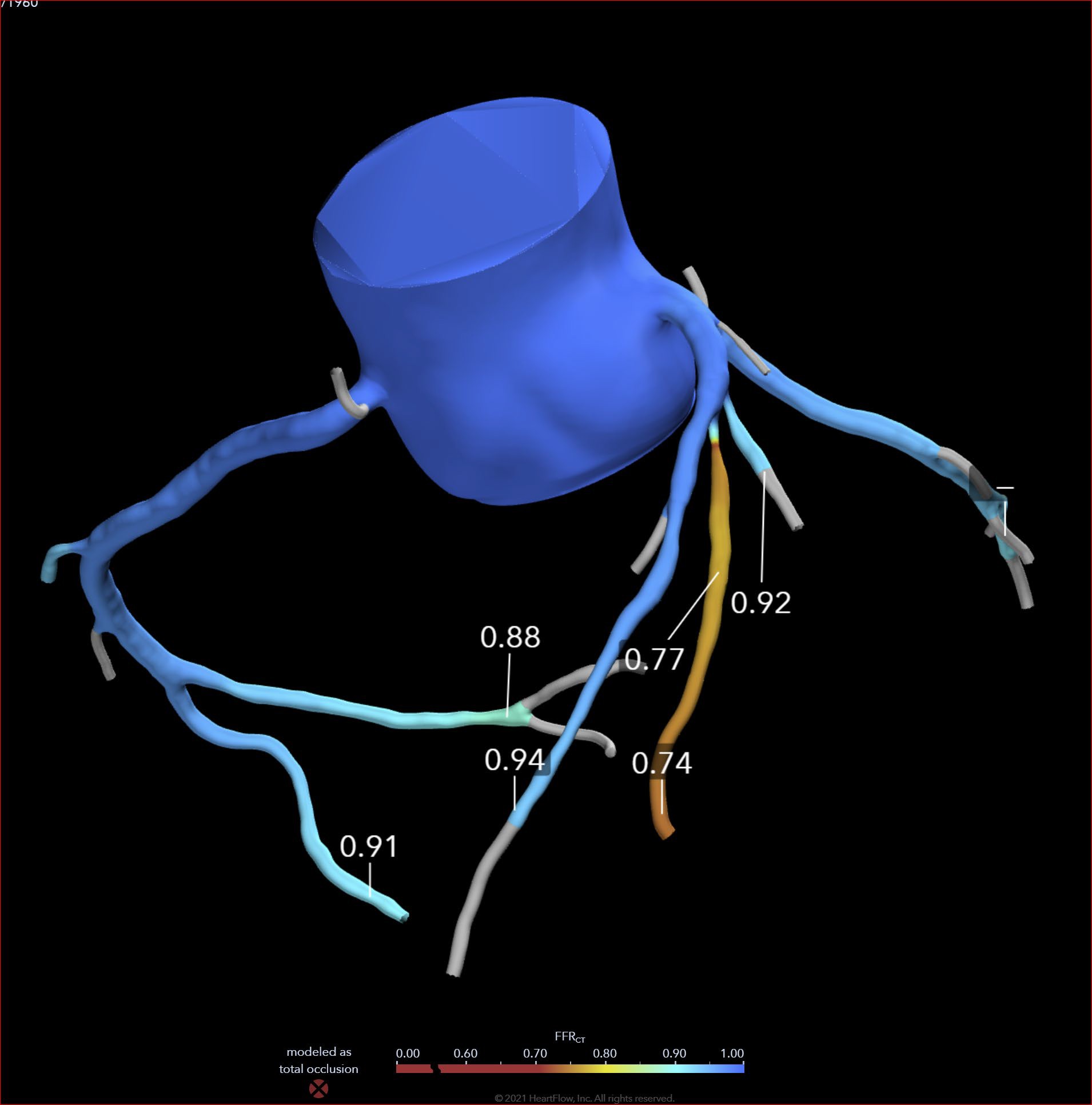

Supplement: Supplementary file 1 — Additional file 1: Figure S1 D1/D2/RI Territory Atherosclerotic Plaque Characteristics. Figure S2 RCA Territory Atherosclerotic Plaque Characteristics. Figure S3 FFR-CT Analysis Based on First CCTA. Figure S4 FFR-CT Analysis Based on Final CCTA. [file 12872_2022_2951_MOESM1_ESM.docx]
